# Pertussis in Afghanistan, 2007–2008 

**DOI:** 10.3201/eid1503.080982

**Published:** 2009-03

**Authors:** Rishtya M. Kakar, Mohammad K. Mojadidi, Jawad Mofleh

**Affiliations:** Shifa College of Medicine, Islamabad, Pakistan (R.M. Kakar, M.K. Mojadidi); Ministry of Public Health, Kabul, Afghanistan (J. Mofleh)

**Keywords:** Disease outbreaks, pertussis, whooping cough, Afghanistan, DEWS, surveillance, immunization, letter

**To the Editor:** Recent reports have raised concerns about transmission of pertussis among troops stationed in Afghanistan and indicated lack of data about pertussis in this country (*1**,**2*). To fill the knowledge gap about pertussis in Afghanistan, we analyzed data collected by the new Disease Early Warning System (DEWS) during Afghan Year 1386, which corresponds to March 21, 2007–March 20, 2008 (*3*) (Figure).

**Figure:**
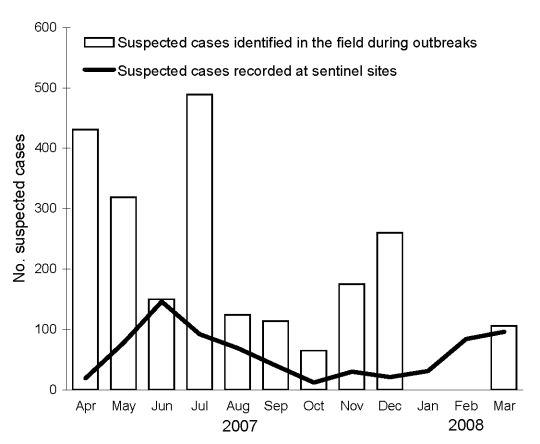
Suspected pertussis cases recorded at sentinel sites and from outbreaks, Afghanistan, April 2007–March 2008.

DEWS collects weekly data on 15 epidemic-prone diseases from 129 sentinel sites, mostly provincial and district hospitals, based on surveillance case definitions of the World Health Organization. The case definition for suspected pertussis is “a person with a cough lasting at least two weeks and having paroxysms of coughing; or inspiratory ‘whoop’; or post-tussive vomiting and without other apparent cause.” During the study period, 718 cases meeting the surveillance definition for suspected pertussis were recorded in weekly reports from patient visits at DEWS sentinel sites.

When a geographic cluster of at least 5 cases of pertussis is suspected in any area, the DEWS team travels to the area and either confirms that cases meet the clinical definition or rejects the outbreak alert. During outbreak response, DEWS personnel record all cases on a linelist (a rough database), collect throat swabs for laboratory culture, treat case-patients and susceptible contacts (infants and pregnant women) with erythromycin, and schedule follow-up vaccination services for the area. During the study period, the team responded to 56 outbreaks of pertussis in the field that involved 2,233 suspected cases and 32 deaths.

Despite difficult geographic features and lack of infrastructure, DEWS collected and transported 203 specimens from patients with suspected pertussis to the Central Public Health Laboratory in Kabul where *Bordetella pertussis* was successfully isolated in bacteriologic culture from 7 (≈3%) of the specimens. This finding compares with that of a recent study from Spain in which 7% of specimens were microbiologically confirmed (*4*).

In 75% of the outbreak areas, vaccination coverage was <50%, and median age of patients with suspected cases was 4 years. Thus, Afghanistan needs to continue its focus on raising immunization coverage to >90% by administering the primary series of diphtheria-pertussis-tetanus vaccine to infants. This effort will help reduce transmission among infants and young children for whom pertussis is most lethal (*5*).

## References

[1] Sagui E, Ollivier L, Gaillard T, Simon F, Brisou P, Puech P, Outbreak of pertussis, Kabul, Afghanistan. Emerg Infect Dis. 2008;14:1173–5. 10.3201/eid1407.071329 18598657 PMC2600351

[2] Cooper NK, Bricknell MC, Holden GR, McWilliam C. Pertussis—a case finding study amongst returnees from Op Herrick. J R Army Med Corps. 2007;153:114–6. 17896541 10.1136/jramc-153-02-09

[3] Disease early warning system. Weekly Morbidity and Mortality Reports 2007–2008, [cited 2008 Dec 3]. Kabul (Afghanistan): Afghan Public Health Institute, Ministry of Public Health. Available from http://www.moph.gov.af/ai-dews-reports/weekly_reports_of_2007/w1-rpt-of-2007/

[4] Vera I, Garcia-Comas L, Ordobas M, Guiterrez A, Sanz JC, Barranco D. Incidence trends in pertussis in the Autonomous Region of Madrid, Spain: 1982–2005. Euro Surveill. 2007;12:E7–8. 17991417 10.2807/esm.12.09.00731-en

[5] Pertussis vaccines: WHO position paper. Wkly Epidemiol Rec. 2005;80:31–9. 15717398

